# Predictors of Acute Myocardial Infraction in Patients With Vasculitis: A Nationwide Inpatient Cross-Sectional Study

**DOI:** 10.7759/cureus.27751

**Published:** 2022-08-07

**Authors:** Gagan Kaur, Avreet S Bajwa, Chia Chi Loh, Sravani Kommuru, Hadia Younis, Yakub Ibrahim, Syed Nurul Aziz, Viralkumar Patel

**Affiliations:** 1 Medicine and Surgery, Sri Guru Ram Das Institute of Medical Sciences and Research, Amritsar, IND; 2 Internal Medicine, Sri Guru Ram Das Institute of Medical Sciences and Research, Amritsar, IND; 3 Internal Medicine, Manipal University College Malaysia, Melaka, MYS; 4 Internal Medicine, Dr. Pinnamaneni Siddhartha Institute of Medical Sciences & Research Foundation, Vijayawada, IND; 5 Medicine, Peshawar Medical College, Peshawar, PAK; 6 Emergency Medicine, Southend University Hospital, Southend-on-Sea, GBR; 7 Internal Medicine, Shaheed Suhrawardy Medical College, Dhaka, BGD; 8 Internal Medicine, Sarasota Memorial Health Care System, Sarasota, USA

**Keywords:** risk-factors, primary percutaneous coronary intervention (pci), diabetes and hypertension, arthopathies, acute myocardial infarcation, coronary vasculitis

## Abstract

Objectives

The primary goal of this study is to explore demographic and comorbid factors that increase the hospitalization risk of acute myocardial infarction (AMI) in patients with vasculitis along with the utilization rate of percutaneous coronary intervention (PCI)/angioplasty. Additionally, we aim to study the prevalence of AMI in vasculitis inpatients based on geographical distribution.

Methods

We conducted a retrospective cohort study using the Nationwide Inpatient Sample (NIS) in 2019 involving 33,210 inpatients hospitalized on emergency-based admissions with a co-diagnosis of vasculitis, subdivided into cohorts without AMI (N = 31,790) and with AMI (N = 1,420) as the primary diagnosis. A binomial logistic regression model was used to evaluate the odds ratio (OR) of predictors associated with AMI in patients with vasculitis compared to the non-AMI cohort.

Results

The prevalence of AMI in the total inpatient population with vasculitis was 4.28%, with a majority of patients being in the older age group of 51-65 years (63%), males (59.2%), and white (59%). Inpatients with vasculitis having pre-existing co-morbid conditions were at greater risk for AMI, such as obesity (OR 2.84, 95%CI 2.78-2.89), metastatic cancer (OR 1.73, 95%CI 1.26-2.37), complicated hypertension (OR 1.64, 95%CI 1.46-1.85), and arthropathies (OR 1.48, 95%CI 1.30-1.68). The in-hospital mortality rate was significantly higher in the AMI cohort compared to the non-AMI cohort (13% vs 2.9%). The utilization rate of PCI/endovascular angioplasty was 13.02% (185 out of 1,420) and had a lower in-hospital mortality rate compared to those managed by medical treatment (8.1% vs 13.8%).

Conclusion

AMI is an important differential diagnosis to consider in vasculitis patients admitted into the hospital with chest pain. Due to the low prevalence of vasculitis and diagnostic challenges, these primary conditions can be often missed. There is a greater risk of inpatient mortality among vasculitis patients with AMI. Therefore, a higher index of suspicion should be exercised, especially in elderly males with risk factors. Vasculitis patients with chronic comorbidities such as arthropathies, obesity and hypertension are at a greater risk for suffering from AMI. Careful screening and management of cardiovascular risk factors is mandatory in vasculitis patients.

## Introduction

Despite the advancement of medical and surgical modalities, cardiovascular disease remains the leading cause of mortality in the United States (US), attributing to 14.2 million deaths in 1999-2020 [1]. Acute myocardial infarction (AMI) carries the highest burden among its complications. Every 40 seconds, a person in the US suffers from a myocardial infarction (MI) [2]. As per the Joint Task Force of the European Society of Cardiology, the American Heart Association (AHA), and the World Heart Federation, AMI is defined as a cardiac injury that presents with elevated cardiac markers detected in AMI ischemia. This universal definition also includes the classification of MI based on etiology and classified as types I-V. Most type-I AMIs are caused by plaque rupture following advanced atherosclerosis. Type-II AMIs are due to coronary artery dissection, coronary artery spasm, and coronary vascular dysfunction, all due to a reduced myocardial perfusion and oxygen demand mismatch [3]. The causes of type-II AMI are vast and not yet fully explored in terms of mortality risk and demographic distribution. Type-I AMI is associated with modifiable risk factors such as smoking, dyslipidemia, diabetes mellitus, and hypertension. On the other hand, type-II AMI risk factors are not restrained by traditional risk factors such as trauma, vasculitis, drug use, anemia, and hyperthyroidism [3].

Coronary artery vasculitis can manifest in the form of AMI. Cardiac involvement in primary vasculitis disorders, although rare, is linked to increased mortality and results in sudden worsening of a patient's prognosis [4-6]. The implication of coronary vessels can be seen in small, medium and large-vessel vasculitis. For example, most cases have been linked to antineutrophil cytoplasmic antibodies (ANCA)-associated systemic vasculitis, Goodpasture syndrome, Wegener's granulomatosis (WG), polyarteritis nodosa (PAN), Kawasaki disease (KD), Takayasu's arteritis (TA), and giant cell arteritis (GCA) [6]. TA is seen in patients under 40 years of age with coronary involvement in 60% of patients, but only 5-20% turn symptomatic. GCA is more common in patients of northern European ancestry, usually over 50 years of age, with low cardiac involvement [4,7]. About 5-20% of patients with PAN have cardiac involvement leading to ischemia and cardiac injury resulting in AMI [4].

A cross-sectional analysis of an inpatient study established that systemic vasculitis is an independent predictor of inpatient mortality due to AMI [8]. Compared to medium and large-vessel vasculitis, small vessel vasculitis is associated with higher odds of mortality-independent of cardiovascular risk factors and cause of AMI [8]. Due to the low prevalence of vasculitis in the general population, risk factors such as age, race, gender, and other influences that typically increase the risk of mortality in AMI patients are not well understood and researched.

In our study, we evaluated the demographic factors and comorbidities that increase the risk of AMI in patients with vasculitis. Additionally, we studied the utilization rate of percutaneous coronary interventions (PCI) and/or endovascular angioplasty for AMI in hospitalized patients with vasculitis, including the cost and length of stay (LOS) of inpatient care. Finally, we examined the geographical distribution of the prevalence of AMI in vasculitis inpatients.

## Materials and methods

Study sample

We conducted a retrospective cohort study using the Healthcare Cost and Utilization Project (HCUP) Nationwide Inpatient Sample (NIS) 2019, the largest healthcare database covering non-federal community hospitals from 48 states in the US. The NIS is publicly available de-identified data. According to the Agency for Healthcare Research and Quality (AHRQ) and the US Department of Health and Human Services, we do not require approval from an institutional review board [9]. We included 33,210 adult inpatients (18-65 years) hospitalized on emergency-based admissions with a co-diagnosis of vasculitis. The study sample was divided by the primary diagnosis of AMI (N = 1,420) versus non-AMI (N = 31,790).

Variables

The variables of interest included demographic characteristics (age, sex, and race) and comorbidities, the co-diagnoses in the patient records, comorbidities such as arthropathies, peripheral vascular disease (PVD), metastatic cancer, diabetes with complications, hypertension (complicated), obesity, hypothyroidism, chronic pulmonary disease, alcohol abuse, depression, and drug abuse. Geographical areas in the NIS dataset are based on the nine census divisions of the US Census Bureau, i.e. New England (Maine, New Hampshire, Vermont, Massachusetts, Rhode Island, and Connecticut), Middle Atlantic (New York, Pennsylvania, and New Jersey), East North Central (Wisconsin, Michigan, Illinois, Indiana, and Ohio), West North Central (Missouri, North Dakota, South Dakota, Nebraska, Kansas, Minnesota, and Iowa), South Atlantic (Delaware, Maryland, District of Columbia, Virginia, West Virginia, North Carolina, South Carolina, Georgia, and Florida), East South Central (Kentucky, Tennessee, Mississippi, and Alabama), West South Central (Oklahoma, Texas, Arkansas, and Louisiana), Mountain (Idaho, Montana, Wyoming, Nevada, Utah, Colorado, Arizona, and New Mexico), and the Pacific (Alaska, Washington, Oregon, California, and Hawaii). The hospitalization outcomes of interest included severity of illness, LOS, total charges, and in-hospital mortality (all-cause). In the NIS, the severity of the condition was measured using the All Patients Refined Diagnosis Related Groups (APR DRG) and classified as minor, moderate, and significant loss of body functioning [9]. The utilization of procedures like PCI and/or endovascular angioplasty during hospitalization was evaluated.

Statistical analysis

We used Pearson's chi-square test and descriptive statistics for categorical data and an independent-sample t-test for continuous data (LOS and total charges) to evaluate the differences between vasculitis patients presenting with AMI vs non-AMI. The analysis included only patients with vasculitis and AMI, which was further stratified by those who received treatment (medical vs PCI/endovascular angioplasty). A binomial logistic regression model was used to evaluate the odds ratio (OR) of predictors associated with AMI in patients with vasculitis compared to the non-AMI cohort (as reference category). A P-value <0.01 was used as a reference to determine the statistical significance test result using IBM SPSS Statistics for Windows, Version 27.0 (Released 2020; IBM Corp., Armonk, New York, US).

## Results

Our study population included 33,210 patients with vasculitis, and the prevalence of AMI was 4.28% (N = 1,420). AMI in vasculitis inpatients was seen in a higher proportion in the age group of 51-65 years (63%), males (59.2%), and whites (59%). There existed a statistically significant difference between AMI and non-AMI cohorts by age and sex (P <0.001) but not by race/ethnicity (P = 0.659). The most prevalent comorbidities seen in the AMI cohort were arthropathies, complicated hypertension, and PVD. And when compared to the non-AMI cohort, there existed a statistically significant higher difference with arthropathies (65.8% vs 57.6%), complicated hypertension (48.6% vs 32.9%), diabetes with complications (24.6% vs 20.8%), and metastatic cancer (3.2% vs 1.8%).

The severity of illness was higher in the AMI cohort with a greater loss of functioning (82.4%) than in non-AMI (63.8%). Vasculitis inpatients with AMI had a higher mean LOS (11.1 days vs 7.8 days) and higher mean total charges ($189,300 vs $108,146) than the non-AMI cohort. The in-hospital mortality rate was significantly higher in the AMI cohort (13% vs 2.9% in non-AMI) among the vasculitis inpatients, as shown in Table 1.

**Table 1:** Differences in demographics and hospital outcomes in vasculitis inpatients AMI: acute myocardial infarction; LOS: length of stay

Variable	AMI (no)	AMI (yes)	Total	P-value
Number of inpatients	31790	1420	33210	-
Age at admission, in %				
18-35 years	19.6	8.5	19.1	<0.001
36-50 years	28.0	28.5	28.1	
51-65 years	52.3	63.0	52.8	
Sex, in %				
Male	42.1	59.2	42.8	<0.001
Female	57.9	40.8	57.2	
Race/ethnicity, in %				
White	58.1	59.0	58.2	0.659
Black	20.0	21.3	20.1	
Hispanic	14.8	11.2	14.6	
Other	7.1	8.6	7.1	
Comorbidities, in %				
Arthropathies	57.6	65.8	57.9	<0.001
Peripheral vascular disease	36.6	35.2	36.6	0.272
Metastatic cancer	1.8	3.2	1.8	<0.001
Diabetes with complications	20.8	24.6	20.9	<0.001
Hypertension, complicated	32.9	48.6	33.6	<0.001
Obesity	22.3	18.0	22.2	<0.001
Hypothyroidism	11.5	7.0	11.3	<0.001
Chronic pulmonary disease	23.6	20.1	23.5	0.002
Alcohol abuse	4.5	4.6	4.5	0.910
Depression	15.2	10.9	15.1	<0.001
Drug abuse	8.1	7.0	8.1	0.152
Severity of illness, in %				
Minor loss of function	7.5	1.4	7.3	<0.001
Moderate loss of function	28.7	16.2	28.2	
Major loss of function	63.8	82.4	64.6	
Other outcomes				
Mean LOS, in days	7.8	11.1	-	<0.001
Mean total charges, in $	108,146	189,300	-	<0.001
In-hospital mortality, in %	2.9	13.0	3.3	<0.001

There existed a directly proportionate relationship between increasing age and the risk of AMI among vasculitis in patients with higher odds seen in the age group of 51-65 years (OR 2.67, 95%CI 2.17-3.28). Males had higher odds (OR 1.68, 95%CI 1.49-1.80) for AMI than females with vasculitis. Among the comorbidities, diabetes (OR 0.99, 95%CI 0.87-1.14), obesity (OR 2.84, 95%CI 2.78-2.89), metastatic cancer (OR 1.73, 95%CI 1.26-2.37), complicated hypertension (OR 1.64, 95%CI 1.46-1.85), and arthropathies (OR 1.48, 95%CI 1.30-1.68) were associated with increased odds for AMI in vasculitis inpatients as shown in Table 2.

**Table 2:** Risk factors for acute myocardial infarction in vasculitis inpatients

Variable	Odds ratio	95% Confidence interval		P-value
		Lower limit	Upper limit	
Age at admission				
18-35 years	Reference			
31-50 years	2.17	1.75	2.70	<0.001
51-65 years	2.67	2.17	3.28	<0.001
Sex				
Female	Reference			
Male	1.68	1.49	1.89	<0.001
Race/ethnicity				
White	Reference			
Black	0.99	0.86	1.16	0.977
Hispanic	0.76	0.63	0.91	0.003
Other	1.28	1.04	1.57	0.018
Comorbidities				
None	Reference			
Arthropathies	1.48	1.30	1.68	<0.001
Peripheral vascular disease	1.10	0.98	1.25	0.118
Metastatic cancer	1.73	1.26	2.37	<0.001
Diabetes with complications	0.99	0.87	1.14	0.948
Hypertension, complicated	1.64	1.46	1.85	<0.001
Obesity	2.84	2.78	2.89	0.002
Hypothyroidism	0.59	0.48	0.74	<0.001
Chronic pulmonary disease	0.79	0.68	0.92	<0.001
Alcohol abuse	0.98	0.75	1.27	0.868
Depression	0.77	0.64	0.92	0.003
Drug abuse	1.04	0.84	1.29	0.698

The utilization rate of PCI/endovascular angioplasty was 13.02% (185 out of 1,420) in the management of AMI in inpatients with vasculitis. PCI/endovascular angioplasty was utilized more in patients in the age group of 51-65 years (70.3%), males (64.9%), and whites (72.2%). The highest considerable utilization was seen in inpatients with diabetes with complications (35.1%), complicated hypertension (64.9%), and hypothyroidism (13.5%). Patients who received PCI/angioplasty had a higher mean total charge ($299,876 vs. $172,395, P <0.001) but there existed no significant difference by the LOS (13.4 days vs. 10.7 days, P = 0.101). Vasculitis in patients with AMI who were managed by PCI/endovascular angioplasty had a lower in-hospital mortality rate than those managed by medical treatment (8.1% vs 13.8%), as shown in Table 3.

**Table 3:** Differences in demographics and hospital outcomes in vasculitis inpatients with acute myocardial infarction PCI: percutaneous coronary interventions; LOS: length of stay

Variable	PCI/Endovascular angioplasty		Total	P-value
	No (-)	Yes (+)		
Number of inpatients	1235	185	1420	-
Age at admission, in %				
18-35 years	9.3	2.7	8.5	0.006
36-50 years	28.7	27.0	28.5	
51-65 years	61.9	70.3	63.0	
Sex, in %				
Male	58.3	64.9	59.2	0.090
Female	41.7	35.1	40.8	
Race/ethnicity, in %				
White	56.9	72.2	59.0	0.011
Black	22.8	11.1	21.3	
Hispanic	11.2	11.1	11.2	
Other	9.1	5.6	8.6	
Comorbidities, in %				
Arthropathies	65.2	70.3	65.8	0.174
Peripheral vascular disease	35.2	35.1	35.2	0.981
Metastatic cancer	3.2	2.7	3.2	0.698
Diabetes with complications	23.1	35.1	24.6	<0.001
Hypertension, complicated	46.2	64.9	48.6	<0.001
Obesity	18.6	13.5	18.0	0.091
Hypothyroidism	6.1	13.5	7.0	<0.001
Chronic pulmonary disease	20.2	18.9	20.1	0.675
Alcohol abuse	4.9	2.7	4.6	0.191
Depression	10.9	10.8	10.9	0.961
Drug abuse	7.7	2.7	7.0	0.006
Severity of illness, in %				
Minor loss of function	1.2	2.7	1.4	<0.001
Moderate loss of function	13.8	32.4	16.2	
Major loss of function	85.0	64.9	82.4	
Other outcomes				
Mean LOS, in days	10.7	13.4	-	0.101
Mean total charges, in $	172,395	299,876	-	<0.001
In-hospital mortality, in %	13.8	8.1	13.0	0.033

The highest prevalence of AMI with vasculitis was seen in the pacific region of the US (17.3%), whereas the lowest prevalence of AMI in vasculitis inpatients was seen in the mountain region, as shown in Table 4.

**Table 4:** Geographical prevalence of acute myocardial infarction with vasculitis in the United States

Geographical Region	Prevalence (%)
New England	5.3
Middle Atlantic	11.3
East North Central	16.9
West North Central	9.9
South Atlantic	16.2
East South Central	6.3
West South Central	12.7
Mountain	4.2
Pacific	17.3

## Discussion

Our study found that 63% of the inpatients with vasculitis who developed AMI were in the age group of 51-65 years. The prevalence of vasculitis is known to be at both ends of the spectrum in terms of age [10]. The increasing risk of AMI in the older age group can be attributed to other co-morbid conditions and superimposing modifiable risk factors for acute coronary syndrome. An important differential diagnosis to consider in younger patients with AMI with unexplained risk factors is primary and secondary vasculitis [11]. A model to predict cardiovascular complications in newly diagnosed WG and microscopic polyangiitis concluded that elderly patients are at higher risk for developing cardiovascular disease in addition to the male gender [12].

Vasculitides such as PAN, KD, TA, and GCA involve the inflammation of the coronary arteries and have been increasingly associated with the incidence of AMI [13]. However, due to limitations in our sample data, we could not analyze each type of vasculitis in vasculitis inpatients and their association with AMI. Tsoukas, et al. from Canada found that 12% of their studied patients with PAN developed AMI and 3% with granulomatosis polyangiitis developed AMI [14]. They also established higher AMI incidence in older age groups with PAN [14]. 

In patients with vasculitis, increased development of AMI has been linked to multiple risk factors such as hypertension and rheumatoid arthritis (RA) [15]. Our risk factor analysis revealed patients with vasculitis and complicated hypertension were 1.5 times more likely to suffer from AMI, which is suspected due to a direct leukocyte invasion of the blood vessel wall causing systemic inflammation and accelerated atherosclerosis. Hypertension causes exacerbation of vascular dysfunction and scarring of renal vessels causing renovascular hypertension. This phenomenon is also seen in TA, where there is damage to the intimal-medial layer of blood vessels, causing intimal hyperplasia leading to arterial stiffness and the formation of arterial plaques, which are well-known risk factors for AMI [15]. Arthropathies such as RA are linked to an increased risk of mortality from vasculitis, although the pathophysiology is not well known. The etiology is related to an auto-immune process causing widespread inflammation within the vessels and accelerating the process of atherosclerosis, similar to hypertension [16,17]. We found that patients with arthropathies were 1.4 times more likely to suffer from AMI in vasculitis inpatients. Although the in-hospital mortality rate due to AMI is improving, we found a mortality rate of 13% in patients with AMI and vasculitis, compared to 2% in non-AMI patients with vasculitis [18]. This finding calls for further studies to predict the population's demographics at risk of developing AMI [18].

PCI/angioplasty is a minimally invasive procedure where a stent is inserted to allow the revascularization of the occluded vessel. The overall utilization rate of PCI in AMI is around 53% [19]. We found that 13% of AMI patients with vasculitis were managed with PCI. Although the recent AHA guidelines recommend PCI for either a large single vessel involvement or a focal multi-vessel disorder and in the setting of vasculitis, medical immunosuppression is preferred until stabilization of the event [10]. Despite the risks, the use of angioplasty with stenting is increasing in managing large vessel vasculitis. PCI remains the preferred interventional management when there is imminent hypoxia to tissues [20]. As per our study, PCI was associated with lower inpatient mortality when compared to medical treatment for vasculitis in patients with AMI. We were unable to identify conclusive evidence for this finding. However, a recent case report of AMI in a young patient with PAN demonstrated the use of PCI following a high dose of steroid management. It concluded careful monitoring of coronary arteries during the chronic phase of PAN [21]. The study also reported sudden cardiac death of the patent, likely due to stent thrombosis [21]. Given the limited literature, we are unable to determine long-term survival rates after PCI in AMI with vasculitis and this requires more longitudinal studies.

The mean hospital cost in patients was higher in patients who underwent PCI. However, given the heterogeneity in hospital pricing and variation in patient comorbidities, this finding may vary in different regions of the country. We initially suspected that this increased expense was a result of a longer duration of stay; however, no major statistically significant difference was found when comparing the length of stay [22].

Our study results should be considered with some limitations. The NIS treats a wide range of vasculitis as a homogeneous entity, not allowing for subtype analysis and AMI association. As a limitation of a retrospective observational study, we cannot establish a causal relationship between AMI and vasculitis. The dataset may underreport other comorbidities in the patient records. Our study derives its strength from the NIS, which provides a population-based dataset with national representation. The results are sufficiently generalizable to the inpatient population, and there is a low risk of reporting bias since the data codes are independently written by an individual practitioner. Having said that, further studies are needed to evaluate subtype associations of middle vs large vessel vasculitis with risk of AMI, rates of PCI, and inpatient costs.

## Conclusions

AMI is an important differential diagnosis to consider in vasculitis patients admitted into the hospital with chest pain. Due to the low prevalence of vasculitis and diagnostic challenges, these primary conditions can be often missed. There is a greater risk of inpatient mortality among vasculitis patients with AMI. Therefore, a higher index of suspicion should be exercised, especially in elderly males with risk factors. Vasculitis patients with chronic comorbidities such as arthropathies, obesity, and hypertension are at a greater risk of suffering from AMI. Careful screening and management of cardiovascular risk factors should be mandatory in vasculitis patients.
